# Occurence of dentinal defects after root canal preparation with R-phase, M-Wire and Gold Wire instruments: a micro-CT analysis

**DOI:** 10.1186/s12903-017-0387-0

**Published:** 2017-06-02

**Authors:** Marcely Cassimiro, Kaline Romeiro, Luciana Gominho, Andressa de Almeida, Larissa Costa, Diana Albuquerque

**Affiliations:** 1 Department of Operative Dentistry and Endodontics, Dental College of Pernambuco, University of Pernambuco (UPE), Camaragibe, PE Brazil; 2 Department of Odontology, Biological Sciences Unit, Federal University of Campina Grande (UFCG), Campina Grande, PB Brazil; 3 Nuclear Department of Energy, Federal University of Pernambuco (UFPE), Recife, PE Brazil; 4 Avenida General Newton Cavalcanti, 1650, Camaragibe, PE 54753-020 Brazil

**Keywords:** Endodontics, Root canal therapy, Root canal preparation

## Abstract

**Background:**

This study aims to evaluate the frequency of dentinal defects after root canal preparation with the ProTaper NEXT, K3XF and WaveOne GOLD systems using microcomputed tomography.

**Methods:**

Sixty permanent mandibular incisors with a single canal were selected. Inspection of the teeth was performed under a stereomicroscope (15x) to observe the presence of pre-existing cracks and fractures lines. Samples were divided into three experimental groups (*n* = 20): ProTaper NEXT (PTN), K3XF (K3XF) and WaveOne GOLD (WOG). Specimens were scanned through high-resolution microcomputed tomography before and after the preparation of the root canal. Subsequently, all the axial images were examined by two different methods to find possible dentinal defects. Furthermore, an analysis of each millimeter of ten apical millimeters was also performed. The absence or presence of dentinal defects was screened by 3 pre-calibrated blinded examiners.

**Results:**

After analysing all 45,720 slices, dentinal defects were observed in 48,33% (22096 slices). PTN, K3XF and WOG groups represented 11,11% (5079 slices), 17,22% (7873 slices) and 20% (*n* = 9144) of the cross-sectional images, respectively. At 10 apical millimeters (600 slices), 33,33% (200 slices) presented some dentinal defects, representing 7,22% (43 slices), 13,33% (80 slices) and 12,77% (77 slices) of the cross-sectional images in the PTN, K3XF and WOG groups, respectively. All the dentinal defects presented in the postoperative images existed in the images prior to instrumentation.

**Conclusions:**

There was no correlation between the preparation of a root canal using the PTN, K3XF and WOG systems and the formation of new dentinal defects.

## Background

In recent years, several mechanical devices and techniques have been developed to further improve the effectiveness of the instrumentation process and to facilitate root canal preparation. More recently, the WaveOne GOLD instrument was introduced with a new thermal surface treatment in combination with an offset parallelogram-shaped cross section [1] using different file diameters and variable tapers according to the manufacturer. In addition to removing contaminated dentin and modelling the root canal, it is important that the instrument conforms to the natural anatomy to minimize damage to the tooth structure [2].

Such possible damage has the potential to induce vertical root fractures (VRF) due to the application of repetitive tensions, such as mastication [3] and occlusal forces [4]. These defects must be prevented and it is still unknown whether small dentinal defects can lead to root fractures [5].

Many studies using root sectioning and microscopy analysis verified a correlation between the preparation of a root canal using NiTi rotary instruments and the formation of dentinal defects [3, 6–8]. Methodologies using microcomputed tomography (micro-CT) have been recommended, allowing a non-destructive two-dimensional and three-dimensional evaluation of root canal preparation [9]. However, even methodologies based on microcomputed tomographic analysis have presented divergent results when assessing dentinal damage formation [10–13].

To date, no definitive conclusion can be made in terms of the clinical implications of these dentinal defects in long-term situations. Also, it is not known whether the improvements of nickel titanium alloys favors the non-formation of defects. Due to the lack of consensus regarding this subject, the objective of this study was to use micro-CT to analyse the presence of dentinal defects after the preparation of a flat-oval root canal system using the ProTaper NEXT, K3XF and WaveOne GOLD instruments. The null hypothesis was that there would not be formation of dentinal defects in the studied groups.

## Methods

### Sample size calculation

The sample calculation was based on the study of Liu et al. [14], who estimated dentinal defects caused by rotary and reciprocating systems. Using statistical software (Epi Info™ 6 for Windows; Centers for Disease Control and Prevention, Georgia, USA) and a 5% margin of error, a sample size with 80% power would consist of 15 lower incisors. Therefore, a sample consisting of 20 lower incisors per group, with a power of 92.2%, was established.

### Sample selection

This study have been performed in accordance with the Declaration of Helsinki and written consent was obtained from each participant. The present research was approved by the University of Pernambuco Ethics Committee (N:43800815.2.0000.5207).

From a total of 129 teeth, 82 permanent mandibular incisors with straight root canals (<5°) [15] extracted for therapeutic reasons from patients without parafunctional habits and with periodontal problems, aged from 30 to 50 years and of both genders were selected. The curvature angles was chosen on the basis of the initial radiographs by using Image J software version 1.46r (National Institutes of Heath, Bethesda, MD). Teeth were disinfected in 0.1% thymol solution over 24 h and kept in purified filtered water for 30 days, proceeding to micro-CT analysis. Periapical radiographs of the teeth in the buccolingual and mesiodistal directions were obtained. The exclusion criteria were the presence of inflammatory resorption, calcification and samples that had more than one root canal. Inspection under a stereomicroscope (Labomed Luxeo 4D, Los Angeles, CA, USA) was performed with 15x magnification to detect any pre-existing cracks or fracture lines. Teeth that did not match the criteria were excluded. All the laboratory procedures were performed by the same operator.

Coronal portions of the selected teeth were removed with a double-sided diamond disc in low rotation under water refrigeration, leaving samples 13 mm in length. The samples were examined and a glide path with a #10 K-file (Dentsply Tulsa Dental Specialties, Tulsa, OK, USA) was performed. The length of the canal was determined by the insertion of the file until the tip was visible in the apical foramen. The distance between the tip of the file and the reference plane was defined as the length of the canal. The working length (WL) was established by subtracting 1 mm from the canal length.

In order to confirm that the anatomy of the teeth was similar for each group, statistical analysis revealed that the ratio of buccolingual (*P* = 0,874) to mesiodistal (*P* = 0,813) coronal third dimensions was not significantly different (ANOVA comparison test; *P* > 0.05) (Table 1).

**Table 1:** Buccolingual and mesiodistal dimensions of the selected teeth (mm)

Distances	PTN	WOG	K3XF
Mesiodistal distance (mm)	3.74 (0.37)	3.82 (0.44)	3.78 (0.35)
Buccolingual distance (mm)	5.80 (0.46)	5.83 (0.61)	5.77 (0.34)

(SD) Standard Deviation

F (ANOVA) test

The tomograph XT H 225 system (Nikon XT H 225 ST, Nikon Metrology, Tring, Herts, UK) offers a microfocused X-ray source, a large inspection volume, and high image resolution and is capable of ultrafast CT reconstruction [16]. The specimens were scanned under industrial computed tomography with a low isotropic resolution of 60 μm, 70 kV and 114 mA to select samples with Vertucci’s type I anatomical configuration [17]. Sixty mandibular incisors were selected and re-scanned with a high resolution of 14 μm. The scanning was performed according to the following parameters: a rotation of 360° degrees around the vertical axis, a rotation step of 0.5°, a camera exposure time of 500 milliseconds, a frame rate of 5 and a 1-mm thick aluminium filter. Two-dimensional images were generated and reconstructed using the software *CT Pro 3D*, version XT 3.1.3 (Nikon Metrology, Tring, Herts, UK). Subsequently, a three-dimensional reconstruction was performed using the software *VGStudio MAX 2.2* (Volume Graphics GmbH, Heidelberg, Germany). Reconstruction parameters were adjusted to suppress noises using the fine-tuning function following a Gaussian filter (Filter size = 3x3x3), resulting in the acquisition of 700 to 800 slices per tooth.

### Root canal preparation

A thin layer of moulding material made of light body addition silicone was used to simulate the periodontal ligament in acrylic resin blocks.

The root canals were irrigated with 2 mL of 2.5% sodium hypochlorite (NaOCl) solution. The glide path was made with a #10 K-file (Dentsply Maillefer). The experimental groups were prepared with ProTaper NEXT (PTN), K3XF (K3XF) and WaveOne GOLD (WOG) instruments, according to the manufacturer’s recommendations.

### PTN group (*n* = 20)

The root canals were prepared using the instruments X1 (17/0.04) and *X*2 (25/0.06) in sequence in a continuous rotary movement until the WL was reached, and all the canals were instrumented on the buccolingual and mesiodistal extensions. The motor used was a VDW Silver (VDW, Munich, Germany) with 300 rpm and 2Ncm of torque. In-and-out pecking movements were used to prepare the root canal.

### K3XF group (*n* = 20)

The root canals were instrumented using 35/0.04, 30/0.06, and 30/0.04 files until the middle third was reached and a 25/0.06 instrument until reaching the WL. All canals were instrumented on the buccolingual and mesiodistal extensions. The files were used in continuous rotary movement with in-and-out pecking movements, using a VDW Silver motor (VDW) with 300 rpm and 2Ncm of torque.

### WOG group (*n* = 20)

The root canals were prepared with a single WOG Primary (25/0.07) file with reciprocating movements. The preparation was made with in-and-out pecking movements until reaching the WL. All canals were instrumented on the buccolingual extensions with brushing movements. The motor used was the VDW Silver (VDW) with 350 rpm and 5Ncm of torque.

All groups were irrigated with 2 mL of NaOCl after each instrument and recapitulation was proceeded during instrumentation with #10 K-file. At the end of the procedure, a final irrigation was made with 2 mL of 17% EDTA and 2 mL of 2.5% NaOCl. The total volume of NaOCl used during all procedures was 12 mL. Each instrument was used only once. Afterwards, the samples were irrigated with 5 ml of distilled water and micro-CT scanning was performed using the same parameters established previously.

### Dentinal defects evaluation

Based on the divergences of results found in previous studies, the present research used two methods to evaluate the micro-CT images. Initially, a complete evaluation of the cross-sectional images was performed before and after the root canal preparation [11, 12] using the software ImageJ 1.33 (National Institutes of Health, Bethesda, MD). Images before and after root canal preparation were analysed for dentinal defects throughout the tooth. In order to proceed the second analysis, the images were divided in intervals of 1 mm, starting from the apex, analysing and pairing the final 10 mm [10] with ImageJ software. Three pre-calibrated blinded observers endodontists who were experienced in analysing micro-CT images performed the two evaluation processes. In both methods, the images were evaluated twice with a 2-week intermission, and in cases of divergence, they were analysed in groups to reach a consensus [12].

## Results

**Fig. 1:**
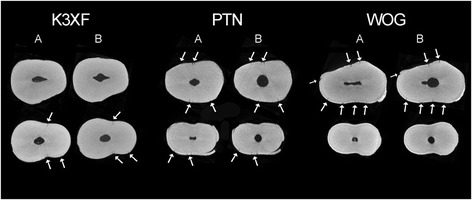
Representative micro-CT cross-section images of mandibular incisors for pre (**a**) and post-instrumentation (**b**)

Of a total of 45,720 slices, 48,33% (22.096 slices) had some dentinal defect. Microcracks were observed in 11,11% (*n* = 5079), 17,22% (*n* = 7873) and 20% (*n* = 9144) of the cross-sectional images in the PTN, K3XF and WOG groups, respectively. Analysing the 10 apical millimeters from a total of 600 slices, 33,33% (200 slices) had some dentinal defect. Microcracks were observed in 7,22% (*n* = 43), 13,33% (*n* = 80) and 12,77% (*n* = 77) of the cross-sectional images in the PTN, K3XF and WOG groups, respectively. All dentinal defects identified in the postoperative scans were already present in the corresponding preoperative images. Thus, no new microcracks were observed after root canal instrumentation with the tested instruments after the analysis (Fig. 1).

## Discussion

The formation of dentinal defects caused by automated preparations of the root canal system has been documented [3, 6, 7, 14, 18, 19]. These studies evaluated the post chemical–mechanical preparation defects through root sectioning and analysis under a stereomicroscope or a scanning electron microscope (SEM). However, studies observed the presence of microcracks also in the control group [19, 20]. In addition, the study by Arias et al. [21] used human jaws of patients aged approximately 82 years old. They performed root sectioning and analysed them under a microscope, which showed pre-existing dentinal defects. De Deus et al. [11] reported that the occurrence of these dentinal damage can be related to the sectioning of the samples. It is known that internal pre-existing defects are impossible to visualize externally under a stereomicroscope and could be due to a patient’s advanced age [21], the tooth extraction process and/or parafunctional habits [22]. In the present study, patients who did not report and did not present any clinical signs of parafunctional habits were selected. Furthermore, the samples were kept hydrated to prevent any roots structural modifications before and in between analysis under micro-CT, which occurred 30 days after their extraction.

Microcomputed tomography (micro-CT) is a highly accurate methodology that does not destroy the sample, allowing three-dimensional visualization by sections before and after preparation of the root canal [10–12] and the effects of it on the anatomy [23]. This technique allows for a quantitative and non-destructive analysis of different variables, such as the volume, surface area, cross section of the model, proportion of the prepared surfaces [24] and formation of dentinal defects [10–12]. This technology allows for the visualization of pre-existing dentinal defects and their precise location before and after the preparation of the root canal, increasing the internal validity of the experiment, as each sample serves as its own control [12]. In the present study, the PTN, K3XF and WOG groups presented dentinal defects on the postoperative images. However, these were coincidental with those in the preoperative images. Thus, it is seen that there is no formation of new defects, only pre-existing dentinal damage, as previous studies reported [11, 12].

With micro-CT methodology, it is possible to analyse various images of the same tooth [12]. In the present study, two forms of analysis were performed to evaluate the formation of dentinal defects after the preparation of a root canal. In both analyses, no new dentinal defects were observed. These results are in agreement with the data obtained by De Deus et al. [11, 12] and contrary to Ceyhanli et al. [10] and Jamleh et al. [13] studies. Higher curvature angles of the roots could explain the increase formation of dentinal defects [10]. This fact can possibly explain the difference of results found in these studies, since higher curvature angle of the roots are found in Ceyhanli et al. [10] study.

The cross-section of the instrument would influence the number of touches on the root dentin, with the potential to promote different degrees of tension. The PTN system has a non-centred rectangular cross section [6] with eccentric movement, minimizing contact with the dentin [25], and two cutting blades. The WOG single file has an offset parallelogram-shaped cross section [1] alternating touches on the dentin with 2 and 1 blades during the 360° spin. The cross sections of these instruments are optimized, and they barely touch the dentin wall during the preparation of the canal. The K3XF instruments have a U-shaped cross section with three cutting blades, three flutes with sinuous profiles and three radial lands [26] which, according to the manufacturer, facilitates centralization of the instrument inside the canal. This transversal section configuration increases the contact with the canal walls compared to the other studied instruments, which could favour the increased formation of dentinal defects. However, there was no formation of dentinal defects after the preparation of the root canal with the three instruments studied.

During the instrumentation of the canal with NiTi rotary systems, a degree of variable rotary forces is applied to the root canal walls and can generate the formation of dentinal defects [27]. The reciprocating movement can prevent this continuous rotary force and constant torque applied to the wall of the root canal, resulting in less dentinal damage [18]. However, studies comparing reciprocating and rotary kinematics report no formation of dentinal defects after the preparation [12, 20, 28]. Even though the WOG file has reciprocating movement and the PTN and K3XF have continuous rotary movement, there was no occurrence of dentinal defects after preparation in the present study.

The conventional NiTi instruments tended to form more dentinal defects than instruments with thermal treatment of the alloy (Memory Wire) [6–8, 18, 19]. However, De Deus et al. [11] and Karatas et al. [18] affirmed that there was no difference between instruments with different thermal treatments, i.e., *R-Phase* and Memory Wire, in the formation of dentinal defects. Considering the studied groups, the different thermal treatments of the NiTi alloys, the PTN system with *Memory Wire,* the WOG system with *Gold Wire* and the K3XF system with *R-Phase™* did not produce different results. The treatments given to the NiTi alloys did not influence the formation of dentinal defects, thus corroborating other studies [12, 20].

The instrument’s taper could be a contributing factor to increasing the tension on the walls of the root canal, consequently developing dentinal defects [27]. However, studies verify that neither the variation of the instrument’s design nor the taper interfere with damage formation [12, 20, 28], as verified in this study. According to De Deus et al. [12], even in instrumented and enlarged canals, this damage is not present.

Analysing the morphology of the lower incisor, the literature reports a deep flattening in the mesiodistal direction of long transversal sections and a larger diameter in the buccolingual direction. Therefore, the preparation was standardized, with all canals instrumented on the buccal and lingual extensions. Furthermore, these root canals have small apical diameters [7], remaining compatible with a preparation size of #25.

The periodontal ligament serves to absorb the tensions of the preparation and limit the movement of the tooth while preventing external forces against the root [29]. However, the preparation of root canals in young pig jaws and posterior root sectioning with microscope analysis showed that the preparation process did not cause any dentinal defect formation, emphasizing the periodontal ligament’s effect on the dissipation of the forces [28]. In the present study, the periodontal ligament was simulated and could have prevented the formation of dentinal defects after the preparation, as observed in various other studies [6, 7, 12, 18]. Studies “in situ” in jaws of human cadavers are suggested, with subsequent analysis under micro-CT to evaluate the possibility of the formation of dentinal defects.

## Conclusions

In view of these results, it is not possible to affirm that dentinal defects were formed after the root canal preparation, independent of the type of instrument used, whether a sequence of rotary instruments or a single reciprocating file and the improvement of the nickel-titanium alloy. Within the limitations of this in vitro study*,* the results can not be directly applied in the clinical situations. However, it is possible to conclude that the preparation of a root canal with ProTaper NEXT, K3XF and WaveOne Gold instruments does not lead to the formation of dentinal defects.

## Abbreviations

Micro-CT: Microcomputed tomography
Mm: Milimeter
SD: Standard deviation
SEM: Scanning electron microscope
WL: Work length
